# Supplementary material for: ACE2 localizes to the respiratory cilia and is not increased by ACE inhibitors or ARBs
Source: Nat Commun. 2020 Oct 28;11:5453. doi: 10.1038/s41467-020-19145-6 (PMC7595232; doi:10.1038/s41467-020-19145-6)
Supplement: Supplementary file 1 — Supplementary Information [file 41467_2020_19145_MOESM1_ESM.pdf]

Supplementary Information for:

ACE2 localizes to the respiratory cilia and is not increased by ACE inhibitors or ARBs

Lee et al.

Supplementary Table 1. Grading of immunohistochemical staining across a human tissue microarray

ACE2 antibodies	Abcam ab15348	Sigma HPA000288	R&D AF933	Abcam ab239924	R&D MAB933	Novus NBP2-67692
Testis	+++	+++	+++	+++	+++	+++
Heart	NS	NS	-	NS	NS	-
Skin	-	NS	-	-	-	-
Seminal vesicle	+++	++	+++	+++	+++	+++
Stomach	NS	NS	NS	-	NS	-
Lung	+	NS	-	-	-	-
Spleen	NS	NS	-	-	-	-
Jejunum	+++	++	+++	+++	+++	+++
Liver	NS	NS	-	-	NS	-
Kidney	++	++	++	+++	+++	+++

Cytoplasmic staining was graded as +++, ++, +, and - according to decreasing intensity. Non-specific stainings were noted as NS. Stains were analyzed and scored by a board-certified pathologist (C.M.S.).

Supplementary Figure 1

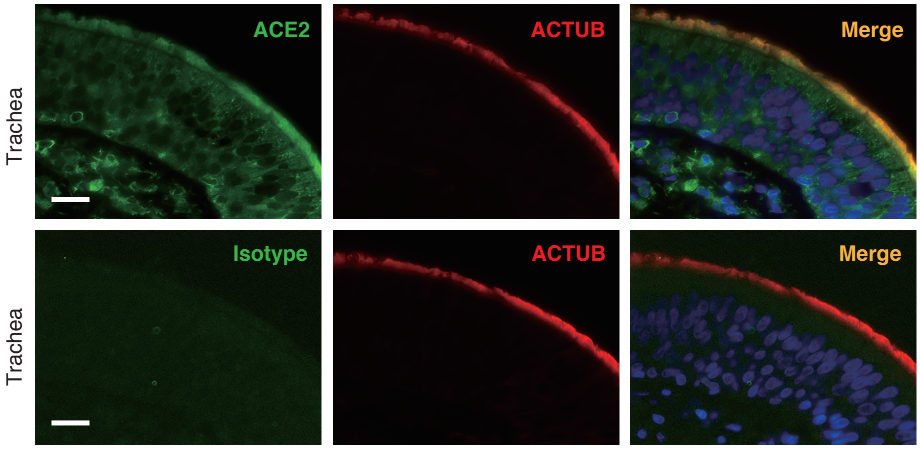

Supplementary Figure 1. ACE2 expression compared to isotype control in human tracheal tissue. Representative immunofluorescence double staining of ACE2 and ACTUB using anti-ACE2 and anti-ACTUB antibodies, respectively, in the top panels compared to double staining of isotype control and ACTUB using rabbit IgG isotype and anti-ACTUB, respectively, in the bottom panels. Nuclei were counterstained using DAPI. Scale bar: 20 µm.
