# Supplementary material for: ACE2 localizes to the respiratory cilia and is not increased by ACE inhibitors or ARBs
Source: Nat Commun. 2020 Oct 28;11:5453. doi: 10.1038/s41467-020-19145-6 (PMC7595232; doi:10.1038/s41467-020-19145-6)
Supplement: Supplementary file 3 — Reporting Summary [file 41467_2020_19145_MOESM3_ESM.pdf]

nature research

Corresponding author(s): Garry Nolan

Last updated by author(s): Aug 25, 2020

Reporting Summary

Nature Research wishes to improve the reproducibility of the work that we publish. This form provides structure for consistency and transparency in reporting. For further information on Nature Research policies, see our Editorial Policies and the Editorial Policy Checklist.

Statistics

For all statistical analyses, confirm that the following items are present in the figure legend, table legend, main text, or Methods section.

n/a	Confirmed	
☐	☒	The exact sample size (n) for each experimental group/condition, given as a discrete number and unit of measurement
☐	☒	A statement on whether measurements were taken from distinct samples or whether the same sample was measured repeatedly
☐	☒	The statistical test(s) used AND whether they are one- or two-sided<br>*Only common tests should be described solely by name; describe more complex techniques in the Methods section.*
☐	☒	A description of all covariates tested
☐	☒	A description of any assumptions or corrections, such as tests of normality and adjustment for multiple comparisons
☐	☒	A full description of the statistical parameters including central tendency (e.g. means) or other basic estimates (e.g. regression coefficient) AND variation (e.g. standard deviation) or associated estimates of uncertainty (e.g. confidence intervals)
☐	☒	For null hypothesis testing, the test statistic (e.g. F, t, r) with confidence intervals, effect sizes, degrees of freedom and P value noted<br>*Give P values as exact values whenever suitable.*
☒	☐	For Bayesian analysis, information on the choice of priors and Markov chain Monte Carlo settings
☒	☐	For hierarchical and complex designs, identification of the appropriate level for tests and full reporting of outcomes
☒	☐	Estimates of effect sizes (e.g. Cohen's d, Pearson's r), indicating how they were calculated

Our web collection on statistics for biologists contains articles on many of the points above.

Software and code

Policy information about availability of computer code

Data collection	The STAnford Research Repository (STARR) Tools, Redcap (9.9.1), and patient's electronic medical records (ex. EPIC) were used to collect patient-related data as permitted by approved Institutional Review Board (IRB) protocols. SlideBook (v6) and Aperio ImageScope (v12.4.3.5008) softwares were used for image-related data collection.
Data analysis	Analyses were carried out using FIJI package of ImageJ 1.52t, IBM SPSS 23 (IBM Corporation, Armonk, NY), and GraphPad Prism 6.0 (GraphPad Software, La Jolla, CA)

For manuscripts utilizing custom algorithms or software that are central to the research but not yet described in published literature, software must be made available to editors and reviewers. We strongly encourage code deposition in a community repository (e.g. GitHub). See the Nature Research guidelines for submitting code & software for further information.

Data

Policy information about availability of data

All manuscripts must include a data availability statement. This statement should provide the following information, where applicable:

- Accession codes, unique identifiers, or web links for publicly available datasets
- A list of figures that have associated raw data
- A description of any restrictions on data availability

The datasets generated during and/or analysed during the current study are available from the corresponding author on reasonable request.

Field-specific reporting

Please select the one below that is the best fit for your research. If you are not sure, read the appropriate sections before making your selection.

- [x] Life sciences
- [ ] Behavioural & social sciences
- [ ] Ecological, evolutionary & environmental sciences

For a reference copy of the document with all sections, see nature.com/documents/nr-reporting-summary-flat.pdf

Life sciences study design

All studies must disclose on these points even when the disclosure is negative.

Sample size	This is a exploratory study using existing tissue specimen during a urgent ongoing COVID-19 pandemic. For this study, sample size of patients were limited by lab environmental biosafety issues, availability of reagents, and the inability to obtain and process additional sinonasal samples during this pandemic. Given the observation nature of our study, we did not perform statistical analyses to predetermine sample size. Sample sizes were chosen by selecting patients whose use of angiotensin receptor blockers (ARBs) or angiotensin-converting enzyme inhibitors (ACEI) could be verified from our sinonasal tissue bank. Control samples were selected by matching covariates as described below. The sample size in this study is comparable to past studies describing ACE2 expression level changes in the heart, kidney, or urine of human or mice subjects taking ARBs or ACEI (Ferrario et al. Circulation 2005, Soler et al. Am J Physiol Renal Physiol 2009, Furuhashi et al. Am J Hypertens 2015)
Data exclusions	Patients whose use or lack of use of ARBs or ACEI cannot be verified by both direct interview and electronic medical/pharmacy records were excluded from the study. Patient tissue samples that did not contain an intact epithelium with well-preserved cilia were excluded. These exclusion criteria were predetermined.
Replication	The three independent cohorts used to compare controls and patients taking ARBs produced similar findings. Results from the Stanford cohort, which contains controls, ARBs, and ACEI experimental groups, were successfully replicated.
Randomization	Participates were allocated into experimental groups based on whether they had been receiving ARBs or ACEI for at least 6 months prior to donation of their sinonasal samples. Participates were allocated into control groups if they have not taken either ARBs or ACEI in the past. Covariates including age, sex, and smoking status were controlled between the experimental and control groups, except for age in the patient cohort from China Medical University as noted in the manuscript.
Blinding	Investigators were blinded to group allocation during immunofluorescent staining of tissues from the three cohorts (Stanford, NTU, CMU) and during the development of the code and the generation of initial datasets. Blinding was not relevant in the exploratory experimental staining of other tissues such as the tissue microarray included in the study.

Reporting for specific materials, systems and methods

We require information from authors about some types of materials, experimental systems and methods used in many studies. Here, indicate whether each material, system or method listed is relevant to your study. If you are not sure if a list item applies to your research, read the appropriate section before selecting a response.

Materials & experimental systems

n/a	Involved in the study	
	x	Antibodies
	x	Eukaryotic cell lines
x		Palaeontology and archaeology
	x	Animals and other organisms
	x	Human research participants
x		Clinical data
x		Dual use research of concern

Methods

n/a	Involved in the study	
x		ChIP-seq
x		Flow cytometry
x		MRI-based neuroimaging

Antibodies

Antibodies used	Rabbit anti-ACE2 (Abcam ab15348; polyclonal; lot GR3333640), rabbit anti-ACE2 (Sigma HPA000288; polyclonal; lot 000009700), goat anti-ACE2 (R&D Systems AF933; polyclonal; lot HOK0320021), rabbit anti-ACE2 (Abcam ab239924; EPR4435(2)), mouse anti-ACE2 (R&D Systems MAB933; 171606), rabbit anti-ACE2 (Novus NBP2-67692; SN0754), mouse anti-acetylated α Tubulin (Santa Cruz sc-23950; 6-11B-1), mouse anti-MUC-1 (NSJ Bio V2372SAF; MUC1/955), rabbit anti-MUC-1 (Abcam ab109185; EPR1023); mouse anti-MUC5AC (Abcam ab212636; 45M1); mouse anti-CD31 (Novus NBP2-47785; C31.3 + C31.7 + C31.10); rabbit anti-CD31 (Abcam ab76533; EPR3094); mouse anti-cytokeratin 8 (Santa Cruz sc-8020), rabbit IgG isotype control (Abcam ab172730; EPR25A), highly cross-adsorbed donkey anti-rabbit Alexa Fluor Plus 647 1:500 (Thermo A32795), highly cross-adsorbed donkey anti-mouse Alexa Fluor Plus 555 1:500 (Thermo A32773).
Validation	For the 6 ACE2 antibodies listed above, aside from the validation that was done by the manufacturers, validation was done by

assessing their staining patterns in tissues and cells known to be ACE2-positive or ACE2-negative. For Rabbit anti-ACE2 (Abcam ab15348; polyclonal; lot GR3333640), additional validation was done by overexpressing the human ACE2 in IMCD3 cells. These were described in the manuscript. All other primary antibodies listed above were validated for immunohistochemistry using Formalin-Fixed Paraffin-Embedded (FFPE) tissues relevant to the protein target of interest. Histologic assessment of staining patterns on relevant tissues and cell types were carefully examined by the co-authors, including a board-certified pathologist. Anti-MUC-1 antibodies listed above stain type II pneumocytes, anti-CD31 antibodies listed above stain endothelial cells, mouse anti-acetylated α Tubulin (Santa Cruz sc-23950; 6-11B-1) stains the motile cilia, mouse anti-MUC5AC (Abcam ab212636; 45M1) stains secretory goblet cells of the airway, and mouse anti-cytokeratin 8 (Santa Cruz sc-8020) stains differentiated cells in the airway epithelia. Rabbit IgG isotype control (Abcam ab172730; EPR25A) did not produce any signal as expected.

Eukaryotic cell lines

Policy information about cell lines

Cell line source(s)	Mouse inner medullar collecting duct cells (ATCC® CRL-2123™) were originally obtained from ATCC.
Authentication	Authentication was provided by ATCC. The mIMCD3 cell line has a very unique cell morphology and is the only known ciliated cell line in our laboratories (and possibly in existence) to have primary cilia facing towards the direction of the air liquid interface (Sang et al. Mapping the NPHP-JBTS-MKS Protein Network Reveals Ciliopathy Disease Genes and Pathways. Cell 2011). Therefore, although we did not perform additional independent authentication of the mouse IMCD3 cell line, based on its unique cell and cilia morphology, we are confident of its authenticity.
Mycoplasma contamination	All cell lines were tested for mycoplasma contamination and found negative.
Commonly misidentified lines (See ICLAC register)	No cell lines used in this study were found in the database of commonly misidentified cell lines that is maintained by ICLAC and NCBI Biosample.

Animals and other organisms

Policy information about studies involving animals; ARRIVE guidelines recommended for reporting animal research

Laboratory animals	Wild-type adult (8-week of age) male C57BL/6J mice were obtained from Jackson Laboratory.
Wild animals	The study did not involve wild animals.
Field-collected samples	The study did not involve samples collected from the field.
Ethics oversight	All animal experiments were conducted in accordance with the institutional AAALAC Guidelines and approved by the Administrative Panel on Laboratory Animal Care (APLAC), Stanford University

Note that full information on the approval of the study protocol must also be provided in the manuscript.

Human research participants

Policy information about studies involving human research participants

Population characteristics	Demographics and covariants of study participants are described in detail in Table 1 of the manuscript. All recruited patients were able to provide written informed consent.
Recruitment	Participants who were undergoing sinonasal surgeries were recruited and consented for donation of sinonasal tissues that would otherwise be discarded. Most of these patients have chronic rhinosinusitis, a non-malignant condition, but patients without a disease process in the sinonasal tissue ("controls") were also recruited. No bias relevant to the aim of the study was present as nasal ciliary ACE2 expression was found not to differ between patients with chronic rhinosinusitis and controls as described in the manuscript. SARS-CoV-2 infected sinonasal tissue was obtained during autopsy with the approval of the patient or relative(s). Given that the SARS-CoV-2 infected nasal tissue was sampled during autopsy of a patient in the advanced, late-stages of COVID-19, the staining characteristics seen may contain inherent artifacts of postmortem tissue preparation and may not be representative of early stages of SARS-CoV-2 binding and cell entry.
Ethics oversight	Studies were approved by the Institutional Review Board (IRB) in accordance with the regulations of the Research Compliance Office at Stanford University, National Taiwan University Hospital, and China Medical University Hospital in Taiwan. SARS-CoV-2 infected sinonasal tissue was obtained with the approval of the ethics commission of Northern Switzerland (EKNZ; study ID: 2020-00969).

Note that full information on the approval of the study protocol must also be provided in the manuscript.
